# The Risk of Malignancies in Celiac Disease—A Literature Review

**DOI:** 10.3390/cancers13215288

**Published:** 2021-10-21

**Authors:** Filippo Pelizzaro, Ilaria Marsilio, Matteo Fassan, Francesco Piazza, Brigida Barberio, Anna D’Odorico, Edoardo V. Savarino, Fabio Farinati, Fabiana Zingone

**Affiliations:** 1 Gastroenterology Unit, Department of Surgery, Oncology and Gastroenterology, University Hospital of Padova, 35128 Padova, Italy; filippo.pelizzaro@unipd.it (F.P.); ilaria.marsilio@unipd.it (I.M.); brigida.barberio@unipd.it (B.B.); anna.dodorico@aopd.veneto.it (A.D.); edoardo.savarino@unipd.it (E.V.S.); fabio.farinati@unipd.it (F.F.); 2 Surgical Pathology and Cytopathology Unit, Department of Medicine (DIMED), University Hospital of Padova, 35128 Padova, Italy; matteo.fassan@unipd.it; 3 Veneto Oncology Institute, IOV-IRCCS, 35128 Padova, Italy; 4 Department of Medicine, Hematology, University Hospital of Padova, 35128 Padova, Italy; francesco.piazza@unipd.it

**Keywords:** celiac disease, malignancy, complication, tumor, lymphoma, carcinoma

## Abstract

**Simple Summary:**

Celiac disease (CeD) is an multiorgan autoimmune disease precipitated by the ingestion of gluten in genetically predisposed individuals. After the initiation of a gluten-free diet, CeD generally has a benign course, with the complete remission of symptoms and a normal life expectancy; however, robust evidence suggests that subjects with CeD are at increased risk of developing malignancies compared to the general population. Peculiar associations with lymphomas, including enteropathy-associated T-cell lymphoma (EATL), and small bowel carcinoma (SBC), as well as correlations with other cancers, have been thoroughly investigated. In this review, we will examine the risk of developing malignancies in patients with CeD, as well as clinical aspects of and therapeutic options for EATL and SBC.

**Abstract:**

Celiac disease (CeD) is an immune-mediated enteropathy precipitated by ingestion of gluten in genetically predisposed individuals. Considering that CeD affects approximately 1% of the Western population, it may be considered a global health problem. In the large majority of cases, CeD has a benign course, characterized by the complete resolution of symptoms and a normal life expectancy after the beginning of a gluten-free-diet (GFD); however, an increased risk of developing malignancies, such as lymphomas and small bowel carcinoma (SBC), has been reported. In particular, enteropathy-associated T-cell lymphoma (EATL), a peculiar type of T-cell lymphoma, is characteristically associated with CeD. Moreover, the possible association between CeD and several other malignancies has been also investigated in a considerable number of studies. In this paper, we aim to provide a comprehensive review of the current knowledge about the associations between CeD and cancer, focusing in particular on EATL and SBC, two rare but aggressive malignancies.

## 1. Introduction

Celiac disease (CeD) is an immune-mediated disease precipitated by the ingestion of gluten in genetically predisposed individuals [1]. Considering that many patients remain undiagnosed for many years before being correctly diagnosed and receiving an appropriate treatment, the exact prevalence of CeD is unknown [2]. According to recently published data, the worldwide serological and histological prevalence rates of CeD are 1.4% and 0.7%, respectively [3]. The prevalence is higher in females compared to males and in children compared to adults. The clinical spectrum of CeD is highly variable, from asymptomatic to severe malabsorptive form [2]. A strict lifelong gluten-free diet (GFD) is the only proven, globally accepted treatment for CeD; however, since it can negatively impact on quality of life, the compliance is often difficult to maintain [4,5].

The great majority of patients respond to a GFD and present an excellent disease prognosis; however, less than 1% of patients develop a series of serious complications that severely affect their survival [6]. Some preneoplastic conditions, such as refractory CeD type 1 and type 2 (RCD-I and RCD-II) and ulcerative jejunoileitis (UJI), as well as several types of malignancies, may complicate the disease course. Among them, non-Hodgkin lymphomas (NHL), including the characteristic enteropathy-associated T-cell lymphoma (EATL) and the small bowel carcinoma (SBC), have been typically associated with CeD [1,7]. In CeD patients, mortality from all causes seems to be 2-fold higher compared to the general population [8,9,10,11,12], and a relevant contribution to this increased death risk is associated with malignant diseases [8,9,10,13]; thus, we aimed to comprehensively review the available evidence regarding the association between CeD and cancer risk. We will focus in particular on lymphomas (among them EATL) and SBC, which are the characteristic malignancies complicating CeD.

## 2. Methods

**Figure 1:**
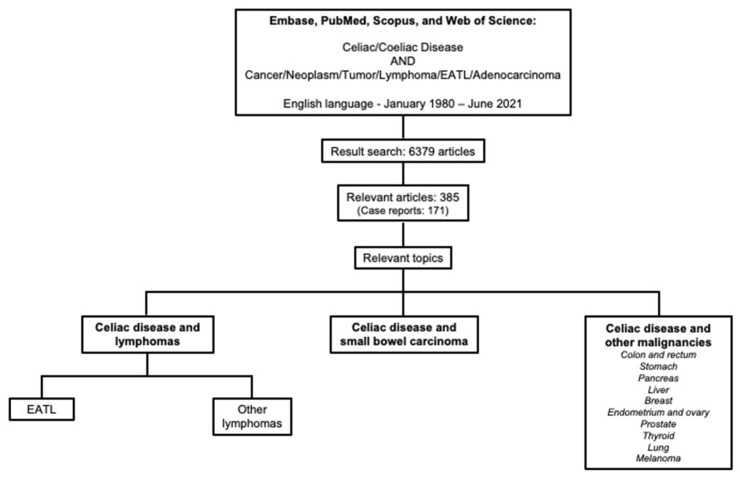
Literature search.

A literature search from the bibliographic databases of Embase, PubMed, Scopus, and Web of Science was performed using the following keywords: (“Celiac Disease” OR “Coeliac Disease”) AND (“Cancer” OR “Neoplasm” OR “Tumor” OR “Lymphoma” OR “EATL” OR “Adenocarcinoma”). Initially, 6379 papers written in English and published between January 1980 and June 2021 were identified. In order to assess the appropriateness for the study’s aim, titles and abstracts of the papers were evaluated by the authors and 385 articles (171 case reports) relating to malignancy in CeD were finally selected and read in full. Paper references were checked to ensure that all potentially relevant articles were retrieved and examined in detail (Figure 1). The articles were categorized by topic and summarized according to their content.

## 3. Celiac Disease and the Overall Risk of Malignancies

The interest in evaluating whether CeD patients have an increased risk of developing malignancies dates back several decades [14,15,16,17,18,19,20,21], and a large number of studies investigating this association have been published so far. Early studies suggested the existence of greater risk, in particular for lymphoma [14,15,16,17,18,19,20,21], while others claimed no differences in comparison to the general population [22]. An increased risk for all cancers (standardized incidence ratio (SIR) = 1.3, 95% CI 1.2–1.5) was reported in a large Swedish population-based cohort study [23]. Interestingly, a decline in the relative risk with the increase in the length of follow-up was observed, with SIR being only slightly and non-significantly elevated (1.1, 95% CI 0.9–1.4) after 10 or more years. These results were confirmed by several other subsequent investigations, although the magnitude of the increased risk was demonstrated to be modest [24,25,26,27,28]. Card et al. [24] found an SIR of 2.0 (95% CI 1.24–3.06) in the peridiagnostic period (<2 years after CeD diagnosis), although this value decreased and became non-significant in the postdiagnostic period (SIR = 1.02, 95% CI 0.70–1.45). The same results were found by West et al. [25]; after excluding the first year after diagnosis of CeD, the overall risk of malignancies was comparable to that of the general population (adjusted hazard ratio (HR) = 1.1, 95% CI 0.87–1.39). Very recently, a large Swedish study involving 47,241 CeD patients confirmed these previous results, demonstrating a very small increase in cancer risk (HR = 1.11, 95% CI 1.07–1.15) compared to controls [28]. This latter study in particular found elevated risk of developing a malignancy only in patients diagnosed after the age of 40 years, and this association was higher in the first year after CeD diagnosis (HR = 2.47, 95% CI 2.22–2.74) but disappeared when the first year of follow-up was excluded (HR = 1.01, 95% CI 0.97–1.05) [28]. According to Grainge et al., the increased risk of developing a malignancy persists up to 15 years after diagnosis and then returns to a similar level to that of non-celiac patients (SIR = 0.92, 95% CI 0.61–1.33, after 15 years of follow-up) [27]. In contrast, other studies showed no differences at all in the risk of developing cancer at all sites between CeD patients and the general population [11,29,30,31,32]. Opposite results were obtained for metanalysis. Han et al. demonstrated an increased risk of malignancies in CeD (odds ratio (OR) = 1.25, 95% CI 1.09–1.44) [33], while Tio et al. claimed no differences compared to the general population (OR = 1.07, 95% CI 0.89–1.29) [12].

Robust data demonstrate that CeD patients have an increased modest long-term risk of mortality from all causes [8,9,10,11,12,13,34]. Corrao et al. [8] showed a 2-fold increase in mortality in their entire CeD patient cohort (standardize mortality ratio (SMR) = 2.0, 95% CI 1.5–2.7); in addition, they demonstrated that SMRs increased significantly in patients with diagnostic delay, in those with severe symptoms at presentation, and in those not adherent to GFD [8]. In a recent population-based cohort Swedish study, including 49,829 patients diagnosed between 1969 and 2017, a small but statistically significant increased mortality risk was demonstrated in CeD patients as compared to the general population [35]. This increased risk of mortality seems to be limited to patients with an overt CeD and not those subjects with positive serology only (unrecognized CeD) [36,37,38]. A relevant proportion of this increased risk of mortality, as reported above, is attributable to the development of malignant diseases [8,9,10,13,34,35]. The SMR for cancers in CeD patients has been shown to vary from 1.61 (95% CI 1.19–2.13) [10] to 2.6 (95% CI 1.7–3.9) [8]. Other studies, while finding no differences in overall cancer-related death, reported significant excess risk of dying from malignant lymphoproliferative diseases in CeD patients [34,39,40].

Despite the conflicting and inconclusive results about the association between CeD and overall cancer risk, the association with some specific malignancies (lymphomas and SBC) is well established.

## 4. Celiac Disease and Lymphomas

Studies evaluating the risk of lymphoproliferative malignancies in CeD patients are depicted in Table 1. Early investigations reported a 100-fold elevation of risk of lymphoma among patients with CeD [41,42], although more recent data have estimated the excess risk of lymphoma to be more modest. Indeed, in these studies, only patients from referral centers and also those with malignancy already present at the time of CeD diagnosis were included. More recent population-based studies, which were not affected by these biases, confirmed the higher risk of developing lymphomas compared to the general population, although standardized incidence ratios of about 3–12 for all NHLs [10,11,23,24,25,26,27,29,31] and 16–40 specifically for gut lymphomas [24,43] have been reported. In a large cohort study conducted in Sweden involving 11,019 patients, the SIR for NHL in CeD patients was reported to be 6.3 (95% CI 4.2–12.5) [23]. Almost equal figures were published by Smedby et al. [44], who confirmed the elevation of NHL risk in CeD (SIR = 6.6, 95% CI 5.0–8.6). Elfstrom and colleagues performed an interesting study involving an impressive number of patients (28,989 with histologically proven CeD (Marsh 3), 13,140 patients with duodenal inflammation (Marsh 1–2), and 3711 patients with positive serology) [45]. While the risk of lymphoproliferative malignancies was increased in CeD patients and in those with inflammation (HR = 2.82, 95% CI 2.36–3.37, and 1.81, 95% CI 1.42–2.31, respectively), in subjects with only positive serology, a risk profile similar to that of the general population (HR = 0.97, 95% CI 0.44–2.14) was reported [45]. Another recent study performed in Sweden confirmed that the risk of lymphoproliferative cancers was higher in CeD patients compared to controls, both considering (HR = 2.20, 95% CI 1.94–2.49) and excluding (HR = 1.75, 95% CI 1.52–2.01) the first year of follow-up after the diagnosis of enteropathy [28]. This association was also confirmed in cohorts of patients with NHL [43,46,47,48,49]. In particular, Gao et al. [46] studied 37,869 NHL patients, finding a risk of CeD more than five times higher (OR = 5.35, 95% CI 3.56–8.06) than that for controls.

The prognosis for CeD patients is severely affected in cases of lymphoma development. Indeed, almost all published studies are concordant in attributing an increased mortality risk from lymphoproliferative diseases in these patients [8,9,10,11,13,37,39,40]. Although the early study by Corrao et al. [8] reported an SMR of 69.3 (95% CI 40.7–112.6) for NHL, subsequent studies scaled back the disease-specific mortality risk. Indeed, an SMR of 11.4 (95% CI 7.8–16.0) was observed in a large (*n* = 10,032) Swedish cohort of hospitalized CeD patients [9], while a rate of 5.07 (95% CI 2.55–10.06) was observed in a population-based cohort study from Finland conducted in biopsy-proven CeD patients [10]. In a competing risk analysis, compared to the general population, CeD patients showed a 0.15% excess risk of dying from NHL up to 10 years postdiagnosis [39].

### 4.1. Enteropathy-Associated T-Cell Lymphoma

Almost a century has passed since the first description in 1937 of the association between small intestinal malignant lymphoma and steatorrhea in 6 patients by Fairley and Mackie [57]. While initially intestinal malabsorption was considered secondary to the presence of lymphoma, a link with CeD was suggested for the first time in the 1960s by Gough et al., who described five cases of small bowel lymphoma in patients with long-standing enteropathy [14]. The term “enteropathy-associated T-cell lymphoma” (EATL) was introduced by O’Farrelly et al. in 1986 and is still widely used to describe the rare form of aggressive T-cell non-Hodgkin lymphoma (NHL) of the small intestine, which characteristically complicates CeD [58].

With an annual incidence of 0.16–1.00 per million people in Western countries, EATL is a rare tumor, accounting for only 5% of all gastrointestinal lymphomas [59,60,61]; however, in parallel to the rising incidence of CeD over the last 40 years [62,63], the incidence of EATL is also progressively increasing [59].

In the vast majority of cases, EATL develops from a complication known as refractory CeD (RCD), which is clinically defined as persistent or recurrent symptoms and signs of malabsorption with villous atrophy, despite a strict GFD for at least 12 months [64,65]. Depending on the absence or presence of aberrant intraepithelial lymphocytes (IELs; lacking surface CD3 and generally CD8 but expressing intracellular CD3), RCD can be classified into two types: RCD type I (RCD-I), which mimes a CeD at diagnosis, and RCD II, which is characterized by the presence of a percentage of aberrant IELs, with monoclonality of TCR in the majority of cases, which is above 20% using flow cytometry and 50% using immunohistochemical analysis [64,65]. Considering that 60–80% of patients with RCD-II will develop intestinal lymphoma within 5 years, this entity can be considered as a prelymphoma (pre-EATL; low-grade lymphoma) [65,66,67,68]. RCD has a very low incidence (0.04–1.5%) [69] but RCD-II represents 15–75% of cases among all patients with RCD according to different studies [66,67,68]. Ulcerative jejunoileitis (UJI) can also complicate CeD, with the development of multiple ulcerations in the intestinal wall evolving in strictures [70,71]. UJI resembles RCD-II in immunological features and both can subsequently progress into EATL through the accumulation in the intestinal epithelium of aberrant (cytoplasmic CD3 9CD3ε0+, CD103+, CD8−, CD4−, TCR-αβ−) and clonal (restricted rearrangements of TCR-γ chain) IELs that are abnormally expanded by the antiapoptotic action of IL-15 [72,73,74]. As far as we know, it is unclear whether RCD-I and RCD-II are different phases of the same process or two different conditions; however, these two entities are unrelated probably, since progression of RCD-I to RCD-II has rarely been described [68] and EALT in patients with RCD-I is an exceptional event [66,75].

Interestingly, it has been hypothesized that viral infections may play a role in the development of CeD complications [76]. Even though Perfetti et al. demonstrated that Epstein–Barr virus (EBV) can be found in 70.5% of biopsies from patients with RCD-I and RCD-II versus 16.6% of controls with uncomplicated CeD [77], data regarding a possible role of this virus in the evolution to EATL are limited and discordant [78,79,80].

According to the World Health Organization Classification of tumors of hematopoietic and lymphoid tissues, EATL can be divided into types 1 and 2 [81,82,83]. EATL type 1 is characterized by non-monomorphic cytomorphology (pleomorphic, anaplastic, immunoblastic), CD56 negativity, and common gains of 1q and 5q regions. It is strongly associated with CeD and HLA-DQ2 genotype. In contrast, EATL type 2, recently named monomorphic epitheliotropic intestinal T-cell lymphoma (MEITL) [84], shows a monomorphic small- to medium-sized tumor cell morphology, frequent CD56 expression and MYC oncogene locus gain, and rare gains of 1q and 5q regions. EATL type 2 is less frequently associated with CeD and HLA-DQ2 genotype [85,86], even if some cases are reported in the literature [87]. EATL type 1 is typically found in Western countries, especially in Northern Europe, where CeD is more prevalent. In Asian populations in contrast, where CeD is rare, intestinal T-cell lymphomas are predominantly if not exclusively type 2 EATL [83,88].

#### 4.1.1. Clinical Presentation

Based on clinical presentation, CeD-associated EATL (EATL type 1) can be classified as primary or secondary. Secondary EATL develops in patients with a known history of CeD, whereas CeD diagnosis is achieved at the time of lymphoma development in patients with primary EATL. Considering the low specificity of symptoms and the low index of clinical suspicion in patients without CeD, the diagnosis of primary EATL is much harder to achieve and typically delayed [89].

The median age at diagnosis of EATL is 60 years, with a comparable proportion of males and females affected [75,83,90]. In patients with secondary EATL, the classical presentation mimics an exacerbation of CeD symptoms, such as abdominal pain, diarrhea, and unexplained weight loss (despite the persistent adherence to GFD) [91]. The concomitant presence of systemic or B symptoms (fever and night sweating; loss of >10% of body weight is present in almost all the patients due to malabsorption) should raise suspicions for this complication, and once the diagnosis is established, they are signs of clinical progression [83,89]. Despite their very low specificity, hypoalbuminemia, anemia, and increased lactate dehydrogenase (LDH) on laboratory tests may also help raise the level of suspicion [91]. Malnutrition is very common, in particular when EATL develops after a long-standing RCD [89,92].

Considering that EATL may be frequently complicated by intestinal perforation, obstruction, and hemorrhage [89,90,93], many of these tumors are diagnosed during emergency laparotomy [75]. At the macroscopic examination, EATL appears as a massive tumor infiltration, which may be transparietal, with thickened plaques, ulcers, or strictures of the intestinal wall [91,94]. The small intestine, and in particular the jejunum, is the most frequent localization of EATL, although this tumor may also arise in the stomach, large bowel, or rectum [83,90,93]. Multiple locations at diagnosis have been frequently reported (50–100%) [90,93,94], and extraintestinal sites may also be involved [83,90,93]. There are some reports of an association between EATL and peripheral eosinophilia [95], mesenteric lymph node cavitation [96], and splenic atrophy [97], which is associated with an increased risk of severe infections and sepsis [98].

#### 4.1.2. Staging and Prognostic Systems

Regarding all lymphomas and EATL, staging procedures are recommended, such as bone marrow examination, abdomen and chest computed tomography (CT), and ultrasound examination of the neck, in order to manage patients appropriately from a therapeutic point of view. In patients with EATL, classic lymphoma staging systems have been demonstrated to be inadequate in prognostic stratification and treatment guidance [99,100]. The accuracy of the well-validated International Prognostic Index (IPI), composed of age, LDH levels, Ann Arbor stage, number of extranodal sites, and Eastern Cooperative Oncology Group performance status (ECOG-PS) [99], turned out to be low for extranodal T-cell lymphomas, including EATL [83,99,101,102,103]. This is mainly because 3 out of 5 components of the IPI (age, number of extranodal sites, and tumor stage) are irrelevant for EATL prognosis. The ability for prognostic discrimination of the Lugano system [100], the staging method commonly used for intestinal lymphomas, is uncertain among EATL patients. The Prognostic Index for Peripheral T-cell Lymphoma (PIT), assessed by age, ECOG-PS, LDH levels, and bone marrow involvement [104], seems to have higher accuracy in predicting survival of EATL patients [83]. Indeed, among the four parameters considered for the prognostic stratification in the PIT model, only bone marrow involvement may be irrelevant for survival prediction of EATL patients, since it is present only in a few subjects [83,105]. More recently, a new clinical prognostic model integrating IPI variables with B-symptoms (fever or night sweats) was developed specifically for EATL patients (EATL Prognostic Index [EPI]) [105]. Three risk groups are identified by the EPI score: patients with B-symptoms (high-risk group: median overall survival [OS] 2 months); patients with an IPI score ≥2 and no B-symptoms (intermediate risk group: median OS of 7 months); patients with an IPI score of 0 or 1 and no B-symptoms (low-risk group: median OS 34 months) [105]. The addition of B-symptoms as a parameter in EPI model resulted in improved recognition of the patients with an extremely poor prognosis needing more aggressive therapies; although very promising, it lacks an independent external validation process. Among the limitations of all these scores is the fact that they have been developed from patient series, which were either very heterogeneous or very small, and in an era preceding the use of intensified chemotherapy or autologous hematopoietic stem cell transplantation (except for EPI study, in which 10 patients were treated with allogenic or autologous stem cell transplantation); thus, these models may be useless in selecting modern therapies.

#### 4.1.3. Imaging and Endoscopy

In cases of suspicion, the presence of an overt lymphoma can be ruled out with several diagnostic tools, including upper and lower endoscopy, chest and abdomen CT scans with enteroclysis, magnetic resonance (MR) enterography, PET scans, wireless capsule enteroscopy, and single- and double-balloon enteroscopy (SBE and DBE). In some cases, laparotomy, intraoperative enteroscopy, and full-thickness biopsies can also be necessary to achieve diagnosis. Nevertheless, the exact diagnostic algorithm in EATL is still unclear [106], mainly because there are no studies directly comparing the accuracy of the different diagnostic modalities [107]. In CT scans, which have higher accuracy compared to small bowel enema, EATL appears as a thickened small bowel loop with possible signs of mucosal ulceration. In addition, CT scans can be preferred allowing an extraintestinal visualization. Indeed, beyond the detection of small bowel thickening, it was proposed that mesenteric lymph node cavitation, intussusception, and small-sized spleen (<120 cm^3^) should raise suspicion for RCD-II or EATL [108]. Although in a prospective cohort of 8 EATL and 30 RCD-II patients 18F-FDG PET demonstrated high sensitivity, with sites affected by the lymphoma (subsequently confirmed histologically) observed in all patients [109], active peristalsis and bowel inflammation may impact its specificity [110]. In the detection of EATL confined to the epithelial layer of the bowel or in the case of multifocality, as well as in assessing the response to treatment, MR enterography has been revealed to be particularly useful [111]. One study showed a sensitivity of 0.88 (95% CI: 0.47, 0.99) and specificity of 0.97 (95% CI: 0.87, 0.99), with an overall high diagnostic accuracy [112].

Diagnostic accuracy can be further improved by endoscopic techniques [113]. Wireless capsule endoscopy enables the exploration of the entire small bowel, assessing the extent of neoplastic involvement and the presence of ulcerations. It is a well-tolerated procedure but it does not allow one to obtain tissue samples and it is contraindicated in cases of suspected strictures [114]; however, it should be the first-line approach to detect complications and to identify patients deserving enteroscopy [115]. DBE, or alternatively SBE, is fundamental in reaching a definite diagnosis of EATL, being the only procedure allowing the collection of biopsy specimens [116].

#### 4.1.4. Pathology

The cornerstone in the diagnosis of EATL remains the histological examination of tumor specimens. EATL type 1 is composed of CD3+, CD4−, CD8−, CD7+, CD5−, and CD56− cells, with a phenotype similar to the majority of normal intestinal intraepithelial T-cell receptor (TCR) α/β+ T lymphocytes [86]. In addition, most of these neoplastic cells express CD30. EATL type 1 originates from cytotoxic IELs (CD8+ αβ intraepithelial T lymphocyte), as demonstrated by the cytotoxic phenotype of tumor cells, which are perforin+, granzyme B+, and TIA-1+ [117,118]. The phenotype mostly consists of medium- to large-sized tumor cells with round or angulated vesicular nuclei, prominent nucleoli, a pale-staining cytoplasm, and an increased mitotic index, which is often associated with moderate to abundant infiltration of eosinophils, histiocytes, and small lymphocytes [94]. The surrounding mucosa shows histologic features of active CeD, such as increased IELs infiltration, crypt hyperplasia, and villous atrophy. EATL type 2, in contrast, is characterized by a monomorphic infiltration of small- to medium-sized T-lymphocytes CD3+, CD4−, CD8+, CD56+, and TCR α/β+ [85,86], and is typically not associated with CeD, suggesting a different pathogenetic mechanism compared to type 1.

It has been speculated that the clonal T-cell population found in the intestinal mucosa of RCD patients could be the precursor of overt EATL [64,119,120]. Indeed, Daum et al. [119] demonstrated that a clonal *TCR-γ* gene rearrangement could be found in 3/8 duodenal biopsies of patients with EATL, in 2/2 patients with UJI, in 2/3 patients with RCD evolving to EATL, and in 1/6 patient with RCD, whereas clonal rearrangements were present in all resected EATL. Although similar results were obtained by Verbeek et al. [121], these authors also demonstrated that flow cytometric determination of aberrant IELs could predict EATL development in RCD patients more accurately than T-cell clonality analysis with polymerase chain reaction (PCR). The presence of *TCR-γ* clonal amplification in the duodenal biopsies of RCD-II and UJI patients, as well as in the subsequent tumor specimens [64,119], and the high predictive value of the quantification of aberrant T-cells for the identification of those RCD patients at risk of evolving in EATL [121], support the concept that these two conditions should be considered as “cryptic lymphomas” and should prompt an extensive diagnostic workup to exclude the presence of malignancy; however, the presence of clonal *TCR-γ* gene rearrangements is not infrequent, even in cases lacking features of RCD-II [122,123]. Hussein et al. demonstrated that *TCR-γ* clonal amplification was present not only in 67% of RCD-II, but also in 17% of patients with RCD-I and in 6% of CeD patients under GFD [122]. Similar results were obtained by Celli et al. [123], who showed clonal T-cell populations in different groups of patients with intestinal lymphocytosis (RCD-I, RCD-II, CeD and Helicobacter pylori-associated lymphocytosis); therefore, for appropriate diagnosis and classification of RCD, the results of *TCR-γ* gene rearrangement analyses should be interpreted cautiously, and immunophenotypic, histological and clinical data should also be considered.

Immunohistochemistry and flow cytometry allow the identification of the abnormal phenotype of IELs in RCD and EATL, which distinguish these two conditions from uncomplicated CeD. Abnormal IELs lose the surface markers CD3, CD4, and CD8, while the expression of intracytoplasmic CD3 (CD3ε) is preserved (in >50% of lymphocytes at immunohistochemistry and >20–25% at flow cytometry) [65,66,124]. According to Verbeek et al., flow cytometric analysis seems to be preferrable to immunohistochemistry in the discrimination of RCD-I and RCD-II using the proposed cut-off of 20% of aberrant IELs [121]. Nevertheless, considering that flow cytometry is available only in tertiary referral centers, commonly both CD3/CD8 immunohistochemistry (simple and accurate in paraffin embedded duodenal tissue at low cost [124]) and *TCR* clonal rearrangement by PCR are used for the evaluation of patients meeting criteria for complicated CeD [125]. Furthermore, the presence of concurrent persistent monoclonality and aberrant immunophenotype (especially if ≥80% CD3ε+ CD8− IELs) is a strong predictor of EATL development, and the continual monitoring of these alterations may be more accurate than snapshot analysis for the evaluation of lymphomagenesis risk [126].

#### 4.1.5. Genetics

Homozygosity for HLA-DQ2 (HLA-DB1*02) and allelic variants of the *MYO9B* gene region have been found to be strongly associated with the development of EATL [127,128]. EATL types 1 and 2 are distinct in their genetic alterations. While increases in chromosomes 1q and 5q occur frequently in EATL type 1 and rarely in EATL type 2, the opposite is true for increases in *MYC* oncogene locus—both types of lymphomas share a high prevalence of 9q gains and 16q losses [86,129,130]; however, these genetic alterations are still not routinely detected for diagnosis, prognostic estimation, and treatment selection.

Very recently, mutational events that drive the progression through lymphoma in CeD patients have been identified in a study involving 50 RCD-II and 19 EATL patients [131]. Gain-of-function mutations in the *JAK1-STAT3* pathway, together with mutations in negative regulators of *NF-kB* (*TNFAIP3* and *TNIP3*), were found to be frequently involved in the emergence of malignant lymphocytes.

#### 4.1.6. Treatment

A standard therapeutic approach in patients with EATL is still lacking. Prospective controlled or randomized clinical trials are difficult to perform because of the low incidence of this malignancy, the wide spectrum of its clinical presentation, its complex diagnosis, and malnutrition or poor performance status, which prevent the possibility of active therapies. Considering the difficulties involved in treatment, these patients have a very poor prognosis, with a 5-year survival rate lower than 20% [66,67,68,75,83,89,93,105,125,132]. A large retrospective study involving 37 patients with EATL revealed that the type of CeD (RCD-I vs. RCD-II), serum albumin levels, completion of at least one cycle of chemotherapy, and surprisingly surgical tumor resection were predictors of overall survival (OS) [75].

The primary role of surgery in the management of EATL is local debulking and resection of tumor masses with a high risk of complication (perforation or bleeding) during chemotherapy and radiotherapy [91]. Moreover, considering that frequently a complication is the revealing event of lymphoma, emergency surgery also has an important diagnostic role [89,90,93]. A major concern when patients are treated surgically is the possible delayed start of chemotherapy; however, data from reports on single cases or small series seems to suggest a better prognosis and a minor risk of perforation in patients with complete resection compared to those with residual disease [90,91,93,133,134]. Interestingly, reductive surgery has also been revealed to be an independent predictor of better prognosis [75].

The most widely used treatment for EATL in clinical practice is standard-dose chemotherapy, although with disappointing results in terms of prognosis (estimated median OS of around 7.5 months), regardless of the stage of the lymphoma at onset [75,89,90,93,135,136,137]. Chemotherapy cannot even be started in more than half of the patients because of their compromised performance status, mainly due to pre-existing unresponsive CeD (with consequent malnutrition), lymphoma dissemination, and in most cases advanced age [91]. Moreover, of those in whom chemotherapy can be started, a further 50% are not able to complete the scheme because of complications, disease relapse, or iatrogenic toxicity [89]. The overall response rate of patients who are able to complete the chemotherapy course is in the range of 40% to 60%, with a slightly higher percentage of responses recorded for early compared to advanced stages [83,89,90,93]. No more than 40% of patients achieve a complete response, with a mean duration of remission in those patients of nearly 6 months [83,89,90,93,136,137]. Only a study from Germany, prospectively evaluating 23 patients with intestinal lymphoma, was able to demonstrate a mean progression-free survival of 28 months (range, 17–39 months) in patients achieving complete remission after chemotherapy and a 2-year survival rate of 28% (95% CI 13–43%) [90]. The cumulative 2-year survival was clearly higher, although not statistically significantly different, in early-stage compared to advanced-stage patients (38% (95% CI 17–59%) vs. 14% (95% CI 0–32%); *p* = 0.13) [90].

Although CHOP (cyclophosphamide, doxorubicin, vincristine, and prednisone) is the most widely used chemotherapy regimen, it provides a 5-year OS of only 9–20% [89,93,135,136,137]. In the large study by Delabie et al. [83], a median OS of 10 months (5-year OS 20%) was reported using a combination chemotherapy containing anthracycline (mainly CHOP; 27/52 patients). Many other therapeutic schemes have been tested, such as BACP (bleomycin, doxorubicin, cyclophosphamide, vincristine, and prednisone) [135], ProMACE-MOPP (prednisone, doxorubicin, cyclophosphamide, etoposide, mechlorethamine, vincristine, and procarbazine) [135], VAMP (vincristine, doxorubicin, high-dose methotrexate, and prednisolone) [89], PEACE-BOM (prednisolone, etoposide, doxorubicin, cyclophosphamide–bleomycin, vincristine, and methotrexate) [89], and CHOEP (CHOP plus etoposide) [136]. In all of these regimens, 2–4 cornerstone drugs, with proven efficacy in lymphoproliferative disease, are present; however, the results are similarly disappointing, with heavier toxicities if the dose intensity is increased (BACOP) or other drugs are included (ProMACE-MOPP, PEACE-BOM, and CHOEP) [91].

The poor survival outcomes achievable with conventional chemotherapy have paved the way for the evaluation of other treatments, such as high-dose chemotherapy followed by autologous stem cell transplantation (ASCT). Unfortunately, this therapeutic option is not available for the majority of patients, who are characterized by poor general conditions, unresponsiveness to debulking therapy, iatrogenic toxicity, or early relapse [91]. Available data regarding ASCT as a treatment for EATL patients are summarized in Table 2.

As far as other novel treatment options are considered, very limited data are currently available. Alemtuzumab, a monoclonal antibody targeting CD52, has been investigated as an adjunctive drug to the CHOP regimen in peripheral T-cell lymphoma [138,139,140]; however, only case reports of EATL patients treated with anti-CD52 have been reported, with conflicting results [141,142,143]. Cladribine (2-CDA), a synthetic purine nucleoside with cytotoxic effects, has been proven to be promising. Furthermore, 2-CDA has been tested more extensively in RCD-II, in which it was demonstrated to improve symptoms and histology, prolong OS, and reduce the probability of EATL development [144,145]. No information is available for overt EATL, except for patients with unresponsive lymphoma treated with 2-CDA in the series by Raderer et al. [133]. Romidepsin, a histone deacetylase inhibitor, was administered as a single agent in a phase II trial on different types of T-cell lymphoma [146]. The single patient with EATL included in this study achieved a complete response, stable 8 months after treatment. Another promising target is represented by IL-15, which has a central role in IEL’s antiapoptotic signaling [74]. A fully humanized IL-15-specific antibody (AMG714) targeting this crucial step of lymphomagenesis has been developed and studies on its activity are ongoing. Finally, brentuximab vedotin, which targets CD30, might be promising when added to conventional chemotherapy and has been suggested as an upfront treatment in EATL [75,147,148].

**Table 2:** Studies investigating treatment with high-dose chemotherapy followed by stem cell transplantation.

Study	Year	Design	No. EATL	No. ASCT	Debulking Chemotherapy	Conditioning Treatment	Response and Survival
Gale et al. [89]	2000	Retrospective	31	2	PEACE-BOM	BEAM	1 CR; disease-free 64 months after diagnosis
Blystad et al. [149]	2001	Retrospective	2 (total 40 NHL)	2	CHOP, MACOP-B	BEAM (or BEAM-like) + TBI	NR
Okuda et al. [150]	2002	Retrospective	1	1	CHOP, ESHAP	MCVC	Relapse after 8 months, death 17 months after ASCT
Chonabayashi et al. [151]	2007	Retrospective	1	1	EPOCH-ICE	MPH-FARA + TBI	Alive at 11 months
Jantunen et al. [152]	2003	Retrospective	5	5	CHOP	BEAM (or BEAM-like)	OS: 2 months. 2 pts died from TRC; 2 progressed early (0 and 1 month); 1 relapsed and died at 14 months
Rongey et al. [153]	2006	Retrospective	1	1	CHOP	BEAM	Alive and in remission at 18 months
Bishton et al. [101]	2007	Retrospective	6	6	IVE + HDMTX	BEAM	5 CR 4 patients alive and in CR after 1.8–4.3 years
Al-Toma et al. [154]	2007	Retrospective	4	4	CHOP	BEAM or MPH-FARA	1 in ongoing CR after 32 months 3 patients died after 2–9 months
Nava et al. [155]	2007	Retrospective	1	1	CHOEP	BEAM	No evidence of residual disease 70 days after ASCT
Reimer et al. [156]	2009	Prospective	5 (total 83 NHL)	5	CHOP + DACMEMP (or PAEM)	TBI + HDCTX	NR
Sieniawski et al. [137]	2010	Prospective	54	14	CHOP—IVE/MTX	TBI + HDMPH or BEAM	Remission rate: 69% Death rate: 39% 5-year OS: 60%
Prochazka et al. [157]	2011	Retrospective	2 (total 29 NHL)	2	PACEB-IVAM-HAM	BEAM	1 CR; survival not reported
Nijeboer et al. [158]	2015	Retrospective	61	8 ^†^	Different regimens	MPH-FARA or CTX-FARA	Complete response: 39% Median OS: 7.4 months 5-year OS: 11%

^†^ 7 underwent autologous stem cell transplantation and 1 allogeneic stem cell transplantation. Abbreviations: PEACE-BOM, prednisolone, etoposide, doxorubicin, cyclophosphamide–bleomycin, vincristine, and methotrexate; BEAM, carmustine, etoposide, cytarabine, and melphalan; EPOCH-ICE, etoposide, prednisone, vincristine, cyclophosphamide and doxorubicin–ifosfamide, etoposide, and carboplatin; MPH-FARA, melphalan + fludarabine; TBI, total body irradiation; CHOP, cyclophosphamide, doxorubicin, vincristine, and prednisone; MACOP-B, methotrexate, doxorubicin, cyclophosphamide, vincristine, prednisone, and bleomycin; NR, not reported; ESHAP, etoposide, methylprednisolone, cytarabine, and cisplatin; MCVC, ranimustine, carboplatin, etoposide, and cyclophosphamide; IVE, ifosfamide, etoposide, and epirubicin; HDMTX, high-dose methotrexate; TRC, transplant-related complications; CHOEP, cyclophosphamide, doxorubicin, vincristine, prednisone, and etoposide; PACEB, doxorubicin, cyclophosphamide, etoposide, bleomycin, vincristine, and prednisone; IVAM, ifosfamide, etoposide, cytosine arabinoside, and methotrexate; HAM, cytarabine and mitoxantrone; HDCTX, high-dose cyclophosphamide; DACMEMP, dexamethasone, carmustine, melphalan, etoposide, and cytarabine; PAEM, etoposide, methylprednisolone, cytarabine, and cisplatin; HDMPH, high-dose melphalan; CTX-FARA, fludarabine, cyclophosphamide.

### 4.2. Other Lymphomas

Several reports demonstrated that CeD patients are at increased risk of developing a wide range of lymphoproliferative diseases, including non-intestinal T-cell lymphomas or B-cell lymphomas. From a public health perspective, considering that EATL is a rare malignancy, this association is more interesting. Indeed, the majority of lymphomas developing in these patients are of the non-EATL type [43,44,48].

In the case–control study by Catassi et al., the risk of NHL as a whole was increased in CeD, although only 1 out of 6 cases of lymphoma was an EATL, while the risk was particularly increased for NHL of T-cell origin and of gastrointestinal localization [43]. Green et al. [29] evaluated a cohort of 381 CeD patients, finding a significantly increased risk of NHL (SMR = 9.1, 95% CI 4.7–13). Among the 9 patients with NHL, three had B-cell lymphomas, four had T-cell lymphomas, and two had large-cell lymphomas that were incompletely classified; five lymphomas were localized in the gastrointestinal tract, three involved lymph nodes, and one involved the skin (mycosis fungoides). EATL was present in only one patient. A subsequent population-based prospective study confirmed that several lymphoma types may develop in CeD patients [44]. In this report, EATL comprised only one-third of NHL and only half of the T-cell lymphomas (SIR for B-cell NHL overall = 2.2, 95% CI 1.3–3.6), while an increased risk of B and T cell lymphomas outside the gastrointestinal tract was also demonstrated (SIR = 3.6, 95% CI 2.3–5.2) [44]. The high risk of B- and T-cell NHL in CeD patients has also been confirmed in more recent data and metanalyses [12,159]; however, not all of the studies are concordant. Recently, van Gils et al., while reporting an increased risk of T-cell NHL in their case–control study (RR = 35.8, 95% CI 27.1–47.4), did not confirm the increased risk for B-cell NHL (RR = 1.4, 95% CI 0.9–2.3) [49].

Considering the relevance of B lymphocytes in the pathogenesis of CeD, the relation between B-cell NHL and CeD, although not completely understood, is not surprising [160]. In particular, the association with CeD with diffuse large B-cell lymphoma has been repetitively reported [47,54,161]. It seems that the link between CeD and lymphoma development is to be found in the chronic inflammation that characterizes these patients. In fact, patients with villous atrophy have a statistically significant higher risk of lymphoproliferative diseases compared to patients with crypt hyperplasia and an increase in intraepithelial lymphocytes [45]. In addition, patients in whom intestinal inflammation does not decrease after GFD (persistent villous atrophy after 6 months of GFD) are at increased risk of lymphoproliferative malignancies compared to those with mucosal healing [55]. Beyond NHL, Hodgkin lymphoma (HL) risk has also been reported as being 2- to 5-fold higher in CeD patients compared to the general population [12,23,31,45].

The risk of developing lymphomas varies according to the small intestinal histopathology [45]. In patients with CeD (Marsh 3), not only was an increased risk of T-cell NHL (HR = 48, 95% CI 15.8–145) confirmed, but associations with and B-cell NHL (HR = 1.9, 95% CI 1.32–2.73) and Hodgkin lymphoma (HR = 2.73, 95% CI 1.26–5.93) were also demonstrated. On the contrary, in patients with Marsh 1 and 2 histology, an association with B-cell NHL (HR = 2.05, 95% CI 1.32–3.18) but not with T-cell NHL (1.36, 95% CI 0.16–11.8) was found [45].

In general, patients with T-cell lymphomas have a poorer prognosis compared to those with B-cell lymphomas [90,159,162]. In a cohort of 63 patients diagnosed with both CeD and lymphoma (gastrointestinal tract in 37% of cases, lymph nodes in 33% of cases, extranodal in 29% of cases), the shortest median survival was demonstrated in patients with T-cell lymphomas other than EATL (10.9 months), while the survival of B-cell lymphoma patients was longer [162]. Similarly, another study found that the mean survival for patients with EATL or non-EATL T-cell NHL was shorter (mean survival periods of 3.2 ± 0.9 years and 2.8 ± 1.2 years, respectively) compared with patients with B-cell NHL or chronic lymphocytic leukemia (mean survival 15.5 ± 2.3 years and 18.9 ± 1.1 years, respectively, *p* = 0.03) [159]. This differential prognosis was also confirmed in a cohort including only intestinal lymphomas [90]: the two-year cumulative survival in patients with B-cell NHL was 94% (95% CI 82–100%) compared to 28% (95% CI 13–43%) in patients with T-cell NHL; cumulative survival rates after two years were similar in EATL and T-cell (non-EATL) patients (28% (95% CI 11.2–44.8%) vs. 29% (95% CI 0–62.5%)) [90].

In contrast, in patients with lymphoproliferative malignancies, the coexistence of CeD does not influence survival. Despite these patients showing an increased risk of death compared to patients with only lymphoproliferative malignancies (adjusted HR = 1.23; 95% CI 1.02–1.48), this greater mortality is only seen in the first year after malignancy diagnosis (adjusted HR = 1.76) and is due to the predominance of T-cell NHL in CeD individuals [163].

## 5. Celiac Disease and Small Bowel Carcinoma

Small bowel carcinoma (SBC) is a remarkably rare neoplasm, which although occurring sporadically in the majority of cases, CeD is recognized as a predisposing condition for this illness [164]. SBC arising with CeD (CeD-SBC) represents a peculiar entity, with several differences in clinical, histopathological, and molecular features compared to sporadic or complicating Crohn’s disease tumor (CrD-SBC).

### 5.1. Epidemiology

SBC are rare malignancies, which although accounting for less than 5% of gastrointestinal cancers, represent around 40% of small bowel neoplasms [165]. The estimated incidence of SBC varies between 3250 and 5300 new cases/year [164,166,167].

The studies investigating the risk of SBC in CeD patients are depicted in Table 3. The first investigations reported a very high relative risk of SBC (19 observed vs. 0.23 expected cases; RR = 82.6) [17], which subsequently has been reduced [23,24,26,29,56,168,169]. A recent Swedish study involving more than 48,000 patients with CeD demonstrated a low absolute risk of SBC, which was significantly increased compared to people without the disease (HR = 3.05, 95% CI 1.86–4.99) [169]. It is thought that SBC develops through the adenoma–carcinoma sequence [170]. Supporting this hypothesis, Emilsson et al. in their study, beyond demonstrating an increased risk of SBC in CeD patients, found a 5.73-fold increased risk of small bowel adenoma (95% CI 3.70–8.88) [169].

The median age at diagnosis of SBC in CeD individuals has been estimated to range from 53 to 62 years, which is lower than that of patients with sporadic SBC (56–72 years) [52,171,172,173,174]. The incidence of SBC cases is double in African Americans (10.2–14.1 per 1,000,000) compared to Caucasians (4.5–7.2 per 1,000,000) [175,176]. The only study investigating ethnic differences in SBC development with CeD reported this tumor only in Caucasians [52].

**Table 3:** Studies evaluating the risk of small bowel carcinoma (SBC) in patients with CeD.

Authors	Year	Country	Design	Size of the CeD Cohort	No. of SBC	Results
Swinson et al. [17]	1983	United Kingdom	Retrospective	235	19	Included only CeD patients with a diagnosed malignancy. Observed cases: 19; expected cases: 0.23. Relative risk = 82.6 Individual risk is extremely low (50 per 100,000/year)
Cottone et al. [50]	1999	Italy	Retrospective	216	1 duodenal adenocarcinoma	NR
Askling et al. [23]	2002	Sweden	Retrospective	Inpatient diagnosed with: CeD 11,019; DH 1354; both diagnoses 226	8 cases in CeD cohort (6 adenocarcinomas, 1 mixed carcinoid-adenocarcinoma and 1 unclassified); no cases in DH cohort; 1 case in CeD + DH cohort	Increased risk in CeD cohort: SIR = 10 (95% CI 4.4–20) No significant increased risk in CeD + DH cohort: SIR = 16 (95% CI 0.4–88)
Green et al. [29]	2003	USA	Prospective	381	3	Cancer diagnosed before or simultaneously CeD Expected cases: 0.1; SMR = 34 (95% CI 24–42)
Card et al. [24]	2004	United Kingdom	Prospective	865	1	Increased risk in the peridiagnostic period (≤2 years after diagnosis of CeD): Crude risk 62 cases/100,000 SIR = 59.97 (95% CI 1.52–334.12) No cases registered in the post diagnostic period (>2 years after diagnosis of CeD)
West et al. [25]	2004	United Kingdom	Retrospective	4732 (23,620 controls)	29 ^†^	Increased risk of gastrointestinal cancers: Overall: aHR = 1.95 (1.27–3.00) First year after diagnosis: aHR = 3.31 (1.40–7.83) Beyond the first year after diagnosis: aHR = 1.65 (0.99–2.76)
Silano et al. [26]	2007	Italy	Prospective	1968	5	Cancer diagnosis preceded diagnosis of CeD. Expected cases: 0.19; SIR = 25 (95% CI 8.5–51.4)
Anderson et al. [30]	2007	United Kingdom	Retrospective	2079 (490 EMA+; 1133 AGA+; 456 AGA+ and EMA-)	3	Despite the increased SIR, no significant association: EMA+: SIR = 23.33 (95% CI 0.00–69.07) AGA+: SIR = 7.28 (95% CI 0.00–21.54) AGA+ and EMA-: SIR = 15.51 (95% CI 0.00–45.90)
Lohi et al. [32]	2009	Finland	Retrospective	6849 (202 tTG positive; 73 EMA positive)	121 ^†^	115 cases in tTG-negative, 6 cases in tTG-positive, 0 cases in EMA-positive. Relative risk for tTG positive patients = 1.38 (95% CI 0.60–3.14)
Grainge et al. [27]	2012	United Kingdom	Retrospective	435	1	Expected cases: 0.09; SIR = 11.1 (95% CI 0.28–61.6)
Elfstrom et al. [168]	2012	Sweden	Prospective	28,882 Marsh score 3 12,860 Marsh score 1–2 3705 positive serology	25 in Marsh 3 24 in Marsh 1–2 4 in positive serology	After 1 year of follow-up after CeD diagnosis: Marsh 3: HR = 2.22 (95% CI 1.19–4.14) Marsh 1–2: HR = 2.49 (1.07–5.79) Positive serology: HR = 4.67 (95% CI 0.53–41.4)
Ilus et al. [56]	2014	Finland	Retrospective	32,439	27	Increased risk of SBC All cases: SIR = 4.29 (95% CI 2.83–6.24) Males: SIR = 3.47 (95% CI 1.66–6.37) Females: SIR = 5.00 (95% CI 2.91–7.99)
van Gils et al. [49]	2018	Netherlands	Retrospective case–control	261/301,337 cases 282/576,971 controls	136 with CeD 5335 without CeD	Increased risk of SBC: RR = 11.9 (95% CI 8.2–17.2)
Caio et al. [177]	2019	Italy	Retrospective	770	5	NR
Emilsson et al. [169]	2020	Sweden	Retrospective	48,119 CeD patients and 239,249 controls	74	Beginning 1 year after diagnosis of CeD, 29 CeD patients (0.06%) and 45 controls (0.02%) developed SBC. HR = 3.05 (95% CI 1.86–4.99) 1 extra case of SBC in every 2944 CeD patients followed for 10 years

^†^ Gastrointestinal cancers, not otherwise specified. Abbreviations: AGA, anti-gliadin antibody; aHR, adjusted hazard ratio; CeD, celiac disease; DH, dermatitis herpetiformis; EMA, endomysial antibody; HR, hazard ratio; NR, not reported; RR, relative risk; SBC, small bowel carcinoma; SIR, standardized incidence rate; SMR, standardized morbidity ratio; tTG, tissue transglutaminase.

### 5.2. Histopathology, Molecular Biology and Pathogenesis

From a histopathological point of view, SBC as a whole are predominantly (52–60%) adenocarcinomas (i.e., of glandular type histology) [178]. This is true also in CeD, where 54% of SBC show a glandular histotype and 15% of cases are instead medullary-type cancers [178]. The majority of CeD-SBC, similarly to sporadic SBC, express intestinal phenotype markers such as the caudal-related homeobox transcription factor (CDX)2, the goblet cell marker mucin (MUC)2, cytokeratin (CK)20, or the small bowel brush border marker CD10 [178].

A high density of CD3+ and CD8+ tumor-infiltrating lymphocytes (TILs) has been demonstrated in CeD-SBC, while lower levels of TILs have been found in sporadic SBC or Crohn’s-disease-associated SBC (CrD-SBC) [171]. This finding suggests a greater host immune response against tumors in CeD-SBC compared to other forms of small bowel tumors, and may contribute to explaining the better prognosis of these patients [52,172,177]. In addition, MSI also induced the antitumor response, which has been claimed as explaining the better survival of CeD-SBC. Indeed MSI, which is a consequence of defective DNA mismatch repair, is found at a higher prevalence rate in CeD-SBC (65–73%) compared to CrD-SBC (0–16%) and sporadic SBC (9–35%) [171,172,173,179,180,181,182,183]. Recently, two main subtypes of CeD-SBC have been described, the first with MSI and high TILs, and the second showing prominent TGF-β activation [184].

Some genetic alterations have been reported in sporadic SBC. In particular, *TP53* (58%), *KRAS* (53.6%), *APC* (26.8%), *SMAD4* (17.4%), and *PIK3CA* (16%) are commonly mutated [185,186]. *TP53* alterations are crucial in small bowel carcinogenesis, as demonstrated by *TP53* gene product overexpression in about half of the cases of both CeD-SBC and CrD-SBC [171,179,187]. *KRAS* mutations, an early event in the adenoma–carcinoma sequence of colorectal cancer, have been found in 31% of CeD-SBC and 12–43% of CrD-SBC [171,179,183,187,188]. While nonsense *APC* mutations have not been reported, in CeD-SBC promoter hypermethylation is frequently present (in 73% of cases) [173]. The aberrant nuclear expression of *β-catenin* observed in most CeD-SBC and sporadic SBC cases suggests an involvement of the *Wnt/β-catenin* pathway [178,189,190]. In addition, several potentially druggable genetic alterations, such as *HER2* mutations or amplification, have been identified in the majority of SBC [171,185,191].

Since non-familial SBC is a very rare tumor, its pathogenesis is largely unknown. Differing from CrD-SBC and sporadic SBC [178,187,192,193], dysplastic lesions have very rarely been found in CeD-SBC [170,174,178]. Moreover, no dysplasia distant from SBC has been identified in CeD patients [170,178,179,180,183,187]. An inflammation–hyperplasia–dysplasia–carcinoma sequence has been hypothesized to explain the pathogenesis of CeD-SBC [164], although additional studies are necessary to confirm this model of carcinogenesis.

### 5.3. Clinical Presentation and Diagnosis

The duration of CeD prior to SBC diagnosis varies between 1.4 and 17 years [52,171,173,174]. Nevertheless, few cases of SBC diagnosed coincidentally with the underlying enteropathy have been reported [171,173,174].

SBC in CeD is localized preferentially in the jejunum and duodenum. The clinical manifestations of SBC at onset are extremely variable. Frequently, bleeding is present, either occult with anemia and positive fecal occult blood test or overt with melena or hematemesis. Abdominal pain and unexplained weight loss are common. Similarly to EATL, SBC is also frequently complicated, with intestinal obstruction, intussusception, and perforation (in case of locally advanced lesions); consequently, diagnosis occurs during emergency surgery [194]. In patients with an established diagnosis of CeD, all of these symptoms apart from isolated anemia should raise suspicion for SBC and should lead to the initiation of the diagnostic workup. These symptoms are shared with other neoplastic complications of CeD, but some characteristics may be peculiar. EATL should be ruled out firstly when the above-reported symptoms are present, along with diarrhea and fever [91]; UJI has to be considered in the presence of diarrhea and intestinal obstruction [2].

Once SBC is suspected, all CeD patients should undergo an upper gastrointestinal endoscopy that allows the lesion to be found and sampled if located proximally to the ligament of Treitz. Nevertheless, since the majority of SBC cases are located in the jejunum, additional diagnostic tests, such as DBE/SBE, CT, and MRI enterography, are usually needed [195,196]. Additionally, small bowel capsule endoscopy has been reported to be useful in the diagnosis of SBC [196], although some concerns have to be considered in its use, including the impossibility to take biopsies for histologic diagnosis and the risks of capsule retention and of missing the tumor, particularly in cases at proximal sites [164].

### 5.4. Prognosis and Treatment

SBC exhibits a worse prognosis compared to colon adenocarcinoma, which is frequently compared due to its rarity [197]. In a retrospective study involving 217 patients with SBC, the authors demonstrated a median OS of 20 months (95% CI 16–24) and a 5-year OS of 26% [198]. Very similar results (median OS of 20.1 months and 5-year OS rate of 26%) were subsequently reported among 491 SBC, which were mostly sporadic [199]. The asymptomatic nature of the tumor until the late disease is among the primary reasons for this bad outcome, with tumors already metastatic at diagnosis in several cases. Recently, Aparicio et al. [200] reported the survival of a large cohort of patients with SBC (n = 347, 6 of them with CD), differentiating the outcome according to the tumor stage; in patients with locally resected cancer the 5-year OS was 65.9%, while those with metastatic SBC the median OS was 12.7 months (2.2 months in patients receiving BSC only and 14.6 months for patients treated with palliative chemotherapy). In addition to the tumor stage at diagnosis, poor differentiation, positive margins, duodenal localization, male gender, black ethnicity, and older age are also associated with worse outcomes [166,199,200,201].

In addition, the OS of SBC significantly differs according to the predisposing chronic immune-mediated intestinal disorder. The predisposing condition is a stage-independent prognostic factor in patients undergoing surgery [171], with CeD-SBC having a significantly longer survival compared to CrD-SBC (5-year survival rate 64.2–83% vs. 26–38%) and sporadic SBC patients [171,172,177,179,183,200,202,203,204].

Some favorable histological and molecular factors may contribute to explaining the better prognosis of CeD-SBC. Glandular and medullary tumors, the predominant histotype of CeD-SBC, are associated with longer OS [178,204,205]. CeD-SBC is typically associated with high TIL density and MSI-high status [171,172,173], and both factors have been associated with better outcomes [171,178,204,205]. A recent study demonstrated an increased proportion of PD-L1-positive cases in both CeD-SBC and CrD-SBC as compared to sporadic SBC [204]. PD-L1+ tumors showed higher TILs and PD-1+ immune cell density, were more frequently MSI-high cases, and exhibited better outcomes compared to PD-L1-negative cases.

Currently, treatment recommendations in CeD-SBC patients are borrowed from sporadic SBC [206]. Surgical resection with lymphadenectomy is the only recommended curative treatment for SBC without distant metastases and is necessary for long-term survival [194]. While it could be sufficient at stage I, resection should be followed by adjuvant chemotherapy (e.g., FOLFOX, LV5FU2 or fluoropyrimidine) at stage II or III [206]. At stage IV, the only recommended treatment is systemic chemotherapy [206]. Tumor molecular analysis may provide the rationale for the use of targeted therapies. For instance, in several case reports of metastatic SBC, the presence of *KRAS* wild-type has been demonstrated to predict responsiveness to antiepidermal growth factor receptor monoclonal antibodies (cetuximab and panitumumab) alone or combined with chemotherapy [207,208]. In contrast, nine patients with metastatic SBC *KRAS* wild-type showed no response to panitumumab in a phase 2 clinical trial (7 showing progression and 2 showing stable disease) [209]. Although rare in CeD-SBC, *HER-2* amplification has been investigated as a potential therapeutic target [191,210]. In addition, immunotherapy, and in particular PD-1/PD-L1 blockade, could be considered in advanced SBC with MSI (i.e., CeD-SBC [204]), as mismatch repair deficiency has been demonstrated to predict response to anti-PD-1 antibodies [211].

## 6. Celiac Disease and Other Malignancies

Whether CeD patients are at higher risk of developing malignancies other than lymphomas and SBC is still under debate. A large body of evidence regarding the epidemiological link between CeD and a wide spectrum of malignancies has been produced (Table 4).

Studies investigating the association between CeD and the risk of developing gastrointestinal (GI) cancers reported conflicting results [11,24,25,27,28,168]. West et al. [25] found an increased risk of any GI cancer compared to the general population, both in the year after diagnosis of CeD (adjusted HR = 3.31, 95% CI 1.40–7.83) and beyond this period, although in the latter cases the magnitude of risk was lower (adjusted HR = 1.65, 95% CI 0.99–2.76). Similarly, in the Lebwohl et al. study, in which a very large number of CeD patients were included, the risk of GI cancer was increased as compared to the general population (HR = 1.34, 95% CI 1.24–1.45), although it became non-significant excluding the first year of follow-up (HR = 1.05, 95% CI 0.96–1.15) [28]. Indeed, the risk of GI cancers seems to decrease over time [24,168]. A large Swedish cohort study demonstrated that in the first year after duodenal biopsy, CeD patients showed a 5.95-fold increase in the probability of being diagnosed with GI cancer, whereas this risk progressively decreased and become non-significant thereafter [168]. Some of the early risks may be due to confounders, as symptoms of GI cancer might cause investigations leading to a diagnosis of CeD. The progressively decreasing risk was demonstrated not only for GI cancer as a whole, but also for specific types of tumors (esophagus, small intestine, colon and rectum, liver, pancreas) [168]. In particular, while the association with colon cancer was particularly high in the first year (HR = 7.94, 95% CI 5.21–12.1), thereafter this risk decreased and returned to a comparable level to that of the general population [168]. Evidence on the risk of colon cancer in patients with CeD is extensive, albeit not conclusive. Two large population-based cohort studies demonstrated an increased risk of colon cancer but did not provide information regarding the length of follow-up [23,56]. On the contrary, a case–control study performed in the United States found no association with premalignant lesions (colonic adenomas) [212], while several other papers also reported no association [11,26,27,28,29,31,213]. Finally, a multicenter Italian study reported that CeD patients have a significantly lower risk of developing colon carcinoma as compared to the general population (SIR = 0.29, 95% CI 0.07–0.45) [214]. With respect to other GI cancers, conflicting results about the risk in CeD patients have been reported for esophagus [23,27,29,31,49,56,168,213], stomach [11,23,26,28,31,56,168], pancreas [23,28,31,56,168,213], and liver [23,28,56,168] cancers (Table 4). In the meta-analysis performed by Han et al. [33], CeD was associated with a 60% increase in GI cancer risk (pooled OR = 1.60, 95% CI 1.39–1.84), and among single types of tumors, an increased risk was found only for esophageal cancer (pooled OR = 3.72, 95% CI 1.90–7.28).

In their study, Askling et al. reported a significantly decreased risk of breast cancer in women with CeD [23]. This finding was confirmed in many other population-based studies [25,26,31,215], including the one by Ilus et al., encompassing more than 20,000 Finnish CeD females [56]. Very recently, this negative association was also demonstrated in a large Swedish population-based study among 29,381 CeD females (HR = 0.83, 95% CI 0.74–0.92) [28]. The reasons for this repetitively reported protective effect of CeD toward breast cancer are not completely clear, although lower BMI and consequently lower estrogen exposure may play a role [216,217]. Moreover, it has been noted that starvation during adolescence and a low growth rate in childhood, both features that can occur in CeD, protect against breast cancer [218,219,220]. Moreover, female patients with CeD showed a significantly decreased risk of endometrial cancer (HR = 0.60; 95% CI 0.41–0.86), while no association was found with ovary tumors (HR = 0.89, 95% CI 0.59–1.34) [215]. Regarding the risk of prostate cancer, one of the most frequent tumors in males, the literature is concordant—CeD patients have a similar risk as the general population [23,25,31,56,221].

Additionally, the associations between CeD and thyroid cancers have been investigated, with conflicting results. In 2006, Kent et al. found a significantly increased risk of thyroid papillary carcinoma in a US cohort of 606 CeD patients, with a very high SIR (22.52, 95% CI 14.90–34.04) [222]. These results were later confirmed in an Italian multicenter cohort study, although the risk was lower and not statistically significant (SIR = 2.55, 95% CI 0.83–5.55) [223]. Other authors came to opposite conclusions. Askling et al. found only 1 case of thyroid cancer among 11.019 CeD patients (SIR = 0.6, 95% CI 0.0–3.3) [23], while 7 cases out of 29,074 CeD patients were detected by Ludvigsson et al. (HR = 0.6, 95% CI 0.3–1.3) [224].

It is likely that there is no significant association between the presence of CeD and melanoma development. While a study reported a 5-fold increase in the risk of melanoma [29], two large population-based study reported no association [23,225]. In particular, a study performed on 29,000 Swedish subjects demonstrated a risk profile similar to that of the general population [225].

**Table 4:** Studies investigating the risk of developing several types of malignances in CeD patients.

Study	Year	Country	Study Design	CeD Cases—*n*	Cancer Cases—*n*	Main Findings
**Colon and rectum cancer**						
Askling et al. [23]	2002	Sweden	Population-based prospective cohort study	11,019	26	Compared to the general population: Increased risk of colon cancer: SIR = 1.9 (95% CI 1.2–2.8) Similar risk of rectum cancer: SIR = 0.8 (95% CI 0.3–1.6)
Green et al. [29]	2003	USA	Hospital-based prospective cohort study	381	3	No increased risk of colon cancer: SIR = 0.8 (95% CI 0.1–7.2)
Viljamaa et al. [11]	2006	Finland	Population-based prospective cohort study	781	4	No increased risk of colon and rectum cancer: SIR = 1.1 (95% CI 0.3–2.8)
Silano et al. [26]	2007	Italy	Hospital-based prospective cohort study	1968	7	No increased risk of colon cancer: SIR = 1.1 (95% CI 0.68–1.56)
Goldacre et al. [31]	2008	United Kingdom	Hospital-based retrospective cohort	1997	11 colon cancers 4 rectum cancers	No increased risk (excluding cases occurred within the first year after CeD diagnosis): Colon: adjusted Rate Ratio = 1.23 (95% CI 0.61–2.20) Rectum: adjusted Rate Ratio = 1.04 (95% CI 0.28–2.67)
Lebwohl et al. [212]	2010	USA	Retrospective cohort study	180	23	No significant increased risk of colorectal adenomas: OR = 0.75 (95% CI 0.41–1.34)
Landgren et al. [213]	2011	USA	Hospital-based retrospective cohort study	NR	11 colon cancers 9 rectum cancers	No increased risk: Colon: adjusted RR = 0.85 (95% CI 0.47–1.54) Rectum: adjusted RR = 1.29 (95% CI 0.67–2.48)
Grainge et al. [27]	2012	United Kingdom	Population-based retrospective cohort study	435	6	No increased risk of colorectal cancer: SIR = 1.17 (95% CI0.43–2.54)
Elfstrom et al. [168]	2012	Sweden	Population-based retrospective cohort study	28,989	First year of follow-up: 49 colon cancers 14 rectum cancers After 1 year: 88 colon cancers 30 rectum cancers	First year of follow-up: Colon: HR = 7.94 (95% CI5.21–12.1) Rectum: HR = 2.57 (95% CI 1.36–4.86) After 1 year of follow-up: Colon: HR = 1.10 (95% CI 0.87–1.39) Rectum: HR = 0.58 (95% CI 0.40–0.85)
Pereyra et al. [226]	2013	Argentina	Multicenter retrospective case–control study	118	24 polyps 18 adenomas 3 advanced neoplastic lesions	No increased risk compared to controls. Polyps: OR = 1.25 (95% CI 0.71–2.18) Adenomas: OR = 1.39 (95% CI 0.73–2.63) Advanced neoplastic lesions: OR = 1.00 (95% CI 0.26–3.72)
Ilus et al. [56]	2014	Finland	Population-based prospective cohort study	32,439	133 colon cancers 51 rectum cancers	Increased risk of colon cancer: SIR = 1.35 (95% CI 1.13–1.58) No increased risk of rectum cancer: SIR = 0.82 (95% CI 0.61–1.07)
Volta et al. [214]	2014	Italy	Multicenter retrospective cohort study	1757	6	Decreased risk of colon carcinoma compared to the general population: SIR = 0.29 (95% CI 0.07–0.45)
Lebwohl et al. [28]	2021	Sweden	Population-based cohort study	47,241	448	No increased risk of colorectal cancer: HR = 1.06 (95% CI 0.96–1.18)
**Esophagus**						
Askling et al. [23]	2002	Sweden	Population-based prospective cohort study	11,019	6	Increased risk of esophageal cancer: SIR = 4.2 (95% CI 1.6–9.2)
Green et al. [29]	2003	USA	Hospital-based prospective cohort study	381	3	Significantly increased risk of esophageal cancer: SIR = 12 (95% CI 6.5–21)
Goldacre et al. [31]	2008	United Kingdom	Hospital-based retrospective cohort	1997	5	No increased risk (excluding cases occurred within the first year after CeD diagnosis): adjusted Rate Ratio = 2.58 (95% CI 0.84–6.07)
Landgren et al. [213]	2011	USA	Hospital-based retrospective cohort study	NR	11	Significantly increased risk: adjusted RR = 1.86 (95% CI 1.03–3.36)
Grainge et al. [27]	2012	United Kingdom	Population-based retrospective cohort study	435	3	No increased risk: SIR = 2.86 (95% CI 0.59–8.37)
Elfstrom et al. [168]	2012	Sweden	Population-based retrospective cohort study	28,989	First year of follow-up: 4 After 1 year of follow-up: 8	First year of follow-up: HR = 6.17 (95% CI 1.52–25.0) After 1 year of follow-up: HR = 1.21 (95% CI 0.55–2.65)
Ilus et al. [56]	2014	Finland	Population-based prospective cohort study	32,439	22	No increased risk: SIR = 1.47 (95% CI 0.92–2.23)
van Gils et al. [49]	2018	Netherlands	Population-based case–control study	28 CeD patients with esophageal cancer	28,070 patients with esophageal cancer and without CeD	No increased risk of esophageal adenocarcinoma: RR = 1.5 (95% CI 0.8–2.6) Increased risk of esophageal squamous cell carcinoma: RR = 3.5 (95% CI 2.1–5.8)
**Stomach**						
Askling et al. [23]	2002	Sweden	Population-based prospective cohort study	11,019	6	No increased risk: SIR = 0.9 (95% CI 0.3–2.0)
Viljamaa et al. [11]	2006	Finland	Population-based prospective cohort study	781	2	No increased risk: SIR = 1.2 (95% CI 0.2–4.5)
Silano et al. [26]	2007	Italy	Hospital-based prospective cohort study	1968	3	Slightly increased risk: SIR = 3.0 (95% CI 1.3–4.9)
Goldacre et al. [31]	2008	United Kingdom	Hospital-based retrospective cohort	1997	8	No increased risk (excluding cases occurred within the first year after CeD diagnosis): adjusted Rate Ratio = 1.83 (95% CI 0.79–3.62)
Elfstrom et al. [168]	2012	Sweden	Population-based retrospective cohort study	28,989	First year of follow-up: 7 After 1 year of follow-up: 24	First year of follow-up: HR = 1.67 (95% CI 0.66–4.22) After 1 year of follow-up: HR = 1.13 (95% CI 0.72–1.77)
Ilus et al. [56]	2014	Finland	Population-based prospective cohort study	32,439	37	No increased risk: SIR = 0.90 (95% CI 0.63–1.23)
Lebwohl et al. [28]	2021	Sweden	Population-based cohort study	47,241	65	No increased risk: HR = 1.21 (95% CI 0.91–1.61)
**Pancreas**						
Askling et al. [23]	2002	Sweden	Population-based prospective cohort study	11,019	9	No statistically significant increase in risk: SIR = 1.0 (95% CI 0.9–3.6)
Goldacre et al. [31]	2008	United Kingdom	Hospital-based retrospective cohort	1997	2	No increased risk (excluding cases occurred within the first year after CeD diagnosis): adjusted Rate Ratio = 0.57 (95% CI 0.07–2.05)
Landgren et al. [213]	2011	USA	Hospital-based retrospective cohort study	NR	13	Significantly increased risk: aRR = 2.27 (95% CI 1.22–4.23)
Elfstrom et al. [168]	2012	Sweden	Population-based retrospective cohort study	28,989	First year of follow-up: 26 After 1 year of follow-up: 38	First year of follow-up: HR = 10.7 (95% CI 5.77–19.7) After 1 year of follow-up: HR = 1.40 (95% CI 0.97–2.02)
Ilus et al. [56]	2014	Finland	Population-based prospective cohort study	32,439	45	Significantly decreased risk: SIR = 0.73 (95% CI 0.53–0.97). The risk was decreased particularly in females (SIR = 0.59, 95% CI 0.36–0.89)
Lebwohl et al. [28]	2021	Sweden	Population-based cohort study	47,241	152	Significantly increased risk: HR = 2.30 (95% CI 1.87–2.82). A significant increased risk persists even after excluding the first-year of follow-up: HR = 1.66 (95% CI 1.32–2.10)
**Liver**						
Askling et al. [23]	2002	Sweden	Population-based prospective cohort study	11,019	11	Increased risk: SIR = 2.7 (95% CI 1.3–4.7)
Elfstrom et al. [168]	2012	Sweden	Population-based retrospective cohort study	28,989	First year of follow-up: 15 After 1 year of follow-up: 39	First year of follow-up: HR = 6.05 (95% CI 2.96–12.4) After 1 year of follow-up: HR = 1.78 (95% CI 1.22–2.60)
Ilus et al. [56]	2014	Finland	Population-based prospective cohort study	32,439	24	No increased risk: SIR = 0.98 (0.63–1.45)
Lebwohl et al. [28]	2021	Sweden	Population-based cohort study	47,241	115	Significantly increased risk: HR = 1.80 (95% CI 1.44–2.25). A significant increased risk persists even after excluding the first-year of follow-up: HR = 1.61 (95% CI 1.26–2.05)
**Breast**						
Askling et al. [23]	2002	Sweden	Population-based prospective cohort study	11,019	7	Significantly decreased risk: SIR = 0.3 (95% CI 0.1–0.5)
Green et al. [29]	2003	USA	Hospital-based prospective cohort study	381	5	No increased risk: SIR = 1.2 (95% CI 0.2–7.2)
Card et al. [24]	2004	United Kingdom	Population-based prospective cohort study	4732	5	No increased risk: Peridiagnostic period: SIR = 1.26 (95% CI 0.15–4.54) Postdiagnostic period: SIR = 0.59 (95% CI 0.12–1.73)
West et al. [25]	2004	United Kingdom	Population-based cohort study	4732	8	Significantly decreased risk: adjusted HR = 0.31 (95% CI 0.15–0.63). The association remained significant after 1 year of follow-up (0.24, 95% CI 0.10–0.60)
Viljamaa et al. [11]	2006	Finland	Population-based prospective cohort study	781	9	No significant increased risk: SIR = 0.9 (95% CI 0.4–1.7)
Silano et al. [26]	2007	Italy	Hospital-based prospective cohort study	1968	3	Significantly decreased risk: SIR = 0.2 (95% CI 0.04–0.62)
Goldacre et al. [31]	2008	United Kingdom	Hospital-based retrospective cohort	1997	6	Borderline decreased risk: SIR = 0.48 (95% CI 0.17–1.04)
Lohi et al. [32]	2009	Finland	Population-based retrospective cohort study	73 (EMA + subjects)	1	No increased risk: RR = 0.71 (95% CI 0.10–5.07)
Grainge et al. [27]	2012	United Kingdom	Population-based retrospective cohort study	435	5	No significant increased risk: SIR = 0.71 (95% CI 0.23–1.66)
Ludvigsson et al. [215]	2012	Sweden	Population-based retrospective cohort study	17,852	151	Decreased risk: HR = 0.85 (95% CI 0.72–1.01) Excluding the first year of follow-up: HR = 0.82 (95% CI 0.68–0.99)
Ilus et al. [56]	2014	Finland	Population-based prospective cohort study	32,439	239	Significantly decreased risk: SIR = 0.70 (95% CI 0.62–0.79)
Lebwohl et al. [28]	2021	Sweden	Population-based cohort study	47,241	383	Significantly decreased risk: HR = 0.83 (95% CI 0.74–0.92). A significant increased risk persists even after excluding the first-year of follow-up: HR = 0.81 (95% CI 0.72–0.90)
**Endometrium and Ovary**						
Askling et al. [23]	2002	Sweden	Population-based prospective cohort study	11,019	7	No decreased risk of ovary cancer: SIR = 1.3 (95% CI 0.5–2.7)
Ludvigsson et al. [215]	2012	Sweden	Population-based retrospective cohort study	17,852	31 endometrium cancers 27 ovary cancers	Significant decreased risk of endometrial cancer: HR = 0.60 (95% CI 0.41–0.86) No significantly decreased risk of ovary cancer: HR = 0.89 (85% CI 0.59–1.34)
**Prostate**						
Askling et al. [23]	2002	Sweden	Population-based prospective cohort study	11,019	14	No significantly increased risk: SIR = 0.7 (95% CI 0.4–1.2)
West et al. [25]	2004	United Kingdom	Population-based cohort study	4732	6	No significantly increased risk: aHR = 1.05 (95% CI 0.42–2.57)
Goldacre et al. [31]	2008	United Kingdom	Hospital-based retrospective cohort	1997	4	No significantly increased risk: adjusted ratio = 0.67 (95% CI 0.18–1.73)
Ludvigsson et al. [221]	2012	Sweden	Population-based retrospective cohort study	10,995	185	No increased risk: HR = 0.92 (95% CI 0.79–1.08)
Ilus et al. [56]	2014	Finland	Population-based prospective cohort study	32,439	248	No significantly increased risk: SIR = 0.97 (95% CI 0.85–1.09)
**Thyroid**						
Askling et al. [23]	2002	Sweden	Population-based prospective cohort study	11,019	1	No increased risk: SIR = 0.6 (95% CI 0.0–3.3)
Kent et al. [222]	2006	USA	Monocentric retrospective cohort	606	3	Significantly increased risk of thyroid papillary cancer: SIR = 22.52 (95% CI 14.90–34.04)
Volta et al. [223]	2011	Italy	Multicenter retrospective cohort study	1757	6	Increased risk of thyroid papillary cancer, although not statistically significant: SIR = 2.55 (95% CI 0.93–5.55)
Ludvigsson et al. [224]	2013	Sweden	Population-based retrospective cohort study	29,074	7	No increased risk of all thyroid cancers: HR = 0.6 (95% CI 0.3–1.3). No increased risk of papillary thyroid cancer.
**Lung**						
Askling et al. [23]	2002	Sweden	Population-based prospective cohort study	11,019	12	No increased risk: SIR = 1.0 (95% CI 0.5–1.7)
Green et al. [29]	2003	USA	Hospital-based prospective cohort study	381	3	No increased risk: SIR = 0.8 (95% CI 0.1–7.2)
Card et al. [24]	2004	United Kingdom	Population-based prospective cohort study	4732	8	No increased risk: Peridiagnostic period: SIR = 1.35 (95% CI 0.16–4.88) Postdiagnostic period: SIR = 1.51 (95% CI 0.55–3.29)
West et al. [25]	2004	United Kingdom	Population-based cohort study	4732	57	Borderline significant decreased risk: aHR = 0.37 (95% CI 0.13–1.02)
Viljamaa et al. [11]	2006	Finland	Population-based prospective cohort study	781	2	No increased risk: SIR = 0.6 (95% CI 0.1–2.1)
Goldacre et al. [31]	2008	United Kingdom	Hospital-based retrospective cohort	1997	13	No increased risk: adjusted Rate Ratio = 1.07 (95% CI 0.57–1.83)
Grainge et al. [27]	2012	United Kingdom	Population-based retrospective cohort study	435	6	No increased risk: SIR = 0.78 (95% CI 0.29–1.69)
Ilus et al. [56]	2014	Finland	Population-based prospective cohort study	32,439	86	Significantly decreased risk: SIR = 0.60 (95% CI 0.48–0.74). This result was confirmed both in males and females
Lebwohl et al. [28]	2021	Sweden	Population-based cohort study	47,241	196	Statistically significant decreased risk when the first-year of follow-up is excluded: HR = 0.84 (95% CI 0.71–0.99)
**Melanoma**						
Askling et al. [23]	2002	Sweden	Population-based prospective cohort study	11,019	4	No increased risk: SIR = 0.6 (95% CI 0.2–1.7)
Green et al. [29]	2003	USA	Hospital-based prospective cohort study	381	5	Significantly increased risk: SIR = 5.0 (95% CI 2.1–12.0)
Lebwohl et al. [225]	2014	Sweden	Population-based retrospective cohort study	29,028	78	No increased risk: aHR = 0.94 (95% CI 0.73–1.20)

Abbreviations: aHR, adjusted hazard ratio; CeD, celiac disease; CI, confidence interval; HR, hazard ratio; NR, not reported; OR, odds ratio; RR, relative risk; SIR, standardized incidence ratio.

## 7. The Protective Effect of a Gluten-Free Diet

Strict adherence to GFD is of paramount importance in reducing the risk of developing CeD complications, and its protective effect was proven more than 30 years ago [21]. Holmes et al. showed that for CeD patients who have taken a GFD for 5 years or more, the risk of developing cancer for all sites is comparable to that of the general population [21]. This is further demonstrated by the significantly increased mortality risk in non-adherent patients (SMR = 6.0, 95% CI 4.0–8.8) [8]. In recent decades, many studies have addressed this issue, confirming the importance of compliance to a GFD in order to decrease the risk of malignancies [9,11,22,170,227,228]. Compared to controls, the risk of developing lymphoproliferative malignancies is higher in patients with villous atrophy (Marsh score 3) than in those with intestinal inflammation without villous atrophy (Marsh scores 1 and 2) [168]. These data, together with the evidence that patients with persistent villous atrophy after the GFD have an increased risk of hematologic and lymphoproliferative malignancies compared to those with mucosal healing [28,55], further reinforce the importance of dietary adherence in diminishing the risk of life-threatening complications.

GFD seems to also be useful in reducing the risk of SBC and GI cancers. Elfstrom et al. [168] demonstrated that the risk of SBC in CeD patients decreases, although does not disappear, after the first year of follow-up, likely because the adoption of a GFD reduces intestinal inflammation and mucosal damage [229]. The risk of SBC according to the follow-up biopsy in CeD was evaluated in the study by Emilsson et al. [169], who confirmed a strong but non-significant reduction in risk with mucosal healing (HR = 0.18, 95% 0.02–1.61).

The effect of a GFD in preventing or reducing the risk of developing malignancies has not been universally proven. Poor compliance to a GFD was not significantly associated with an increased risk of malignant lymphoma in individuals with CeD diagnosed in adulthood in the study by Olen et al. [228]. Specifically, the risk of T-cell lymphoma (OR = 1.01, 95% CI 0.32–3.15) or intestinal lymphomas (OR = 0.66, 95% CI 0.17–2.56) was not increased in non-compliant patients, while an association with B-cell lymphoma (OR = 4.74, 95% CI 0.89–25.3) and extraintestinal lymphoma (OR = 3.00, 95% CI 0.73–12.29) was demonstrated although did not achieve statistical significance [228]. Other authors, based on the reduced malignancy risk in asymptomatic CeD patients diagnosed because of familiarity as compared to patients diagnosed because of symptoms, claimed that there is no convincing evidence that GFD further reduces the risk of malignant complications in asymptomatic subjects [46]. Nevertheless, on the basis of the majority of the available data and awaiting more conclusive studies, a rigorous and life-long GFD should be maintained in all patients with CeD, regardless of the presence of symptoms.

## 8. Conclusions

It is difficult to draw firm conclusions regarding the association between CeD and the risk of developing neoplasms, mainly due to the largely variable clinical presentation, since these patients can be either asymptomatic or severely symptomatic.

In contrast to the increase in awareness of the pathogenesis of CeD that occurred in the last decade, as well as the reduction of the diagnostic delay, we still have poor knowledge of the risk factors that may contribute to the development of CD-associated neoplasms. Based on the available literature, we can report that CeD patients have a modestly increased risk of developing lymphomas, in particular the characteristic EATL, as well as SBC, as compared to the general population. Several studies have investigated the association of CeD with other cancer types, although so far there is a lack of conclusive evidence to suggest a higher prevalence of other malignancies in CeD patients. On the contrary, the presence of CeD seems to be protective against the development of some tumors, such as breast cancer in women, although the reason why is not completely clear. The majority of data come from adult CeD patients, while very limited data are available on malignancies in children, with conflicting results shown [13,23,27,168]. The study by Solaymani-Dodaran et al. reported increased mortality due to cancer in children with CeD [13], while diagnostic delay was found as a risk factor for increased cancer incidence and increased mortality [8,26]. A subsequent paper from the same research group confirmed that in childhood-diagnosed CeD patients, an increased mortality risk from external causes and lymphoproliferative malignancies persisted after a long follow-up [34].

Overall, the risk of developing EATL and SBC is very small in humans. Despite this, these tumors are burdened by a very poor prognosis, and strategies aimed at reducing their incidence should be followed. Until now, no surveillance programs have been introduced in CeD patients and probably will not be implemented in the future, since the relative rarity of these tumors means that active surveillance is not cost-effective; therefore, even if the effect of a strict GFD in preventing the development of cancer in CeD is still debated, it is the only available preventive strategy able to reduce the risk of these very aggressive forms of cancer. It seems reasonable that chronic inflammation maintained by continuous exposure to dietary gluten leads to persistent activation of immune and inflammatory signals, ultimately favoring the onset or progression of neoplastic foci.

In conclusion, unlike the important steps forward that have been made regarding the knowledge of the pathogenetic mechanisms of CeD in the last few years, as well as in the reduction of diagnostic delay, we still know little about the risk factors and mechanisms that contribute to the development of neoplasms in these patients. Further experimental data and large multicenter cohort studies, conducted not only in Western countries, are needed to ensure better comprehension of the association between malignancies and CeD. In addition, although large phase 3 clinical trials are hard to perform since EATL and SBC are rare, innovative clinical trial designs and multicenter collaborations are crucial to improve the management of patients with these infrequent but devastating tumors.

## Figures and Tables

**Table 1:** Studies investigating the association between CeD and the risk of lymphoma and lymphoma-associated mortality.

Study	Year	Country	Study Design	CeD Cases—*n*	Lymphoma Cases—*n*	Main Findings
Holmes et al. [21]	1989	United Kingdom	Monocentric retrospective cohort study	210	9	Increased risk of NHL: SIR = 42.7 (95% CI 19.6–81.4)
Cottone et al. [50]	1999	Italy	Hospital-based retrospective cohort	228	6	Incidence of NHL was 3/100 against an expected incidence of 0.8 (*p* < 0.01)
Green et al. [51]	2001	USA	Nationwide cross-sectional survey	1612	3	Relative risk for the development of lymphoma of 300 (60–876)
Corrao et al. [8]	2001	Italy	Multicenter prospective cohort study	1072	16 (death from NHL)	Increased risk of death from NHL: SMR = 69.3 (95% CI 40.7–112.6)
Askling et al. [23]	2002	Sweden	Population-based prospective cohort study	11,019	44	Increased risk of: NHL: SIR = 6.3 (95% CI 4.2–125) HL: SIR = 4.6 (95% CI 1.7–10)
Catassi et al. [43]	2002	Italy	Multicenter case–control study	6	653	CeD was associated with: NHL: OR = 3.1 (95% CI 1.3–7.6) Primary gut NHL: OR = 16.9 (95% CI 7.4–38.7) T-cell NHL: OR = 19.2 (95% CI 7.9–46.6)
Peters et al. [9]	2003	Sweden	Population-based retrospective cohort study	10,032	22	Excess of mortality for NHL: SMR = 11.4 (95% CI 7.8–16.0)
Green et al. [29]	2003	USA	Hospital-based prospective cohort study	381	9	Increased risk of NHL: standardized morbidity ratio = 9.1 (95% CI 4.7–13).
Howdle et al. [52]	2003	United Kingdom	Clinical registry-based cohort	37	86	37/86 with lymphoma
Card et al. [24]	2004	United Kingdom	Population-based prospective cohort study	869	12	Peridiagnostic period: NHL: SIR = 20.94 (95% CI 6.8–48.86) Small bowel NHL: SIR = 358.8 (95% CI 74.01–1048.34) Postdiagnostic period: NHL: SIR = 5.8 (95% CI 1.58–14.86) Small bowel NHL: SIR = 40.51 (95% CI 1.03–225.68)
West et al. [25]	2004	United Kingdom	Population-based cohort study	4732	23	Increased risk of lymphoproliferative disease: aHR = 4.3 (95% CI 2.4–7.7)
Farré et al. [53]	2004	Spain	Multicenter case–control study	5	298	No risk of lymphoma detected in silent or recognized CeD patients: OR = 0.62 (95% CI 0.10–3.79)
Smedby et al. [44]	2005	Sweden	Population-based prospective cohort study	11,650	56	Increased risk of: NHL overall: SIR = 6.6 (95% CI 5.0–8.6) B-cell NHL: SIR = 2.2 (95% CI 1.3–3.6) T-cell NHL: SIR = 51 (95% CI 35–68) Intestinal NHL: SIR = 24 (16–35) Non-intestinal NHL: SIR = 3.6 (2.3–5.2) No increased risk of HL: SIR = 1.0 (95% CI 0.02–5.6)
Viljamaa et al. [11]	2006	Finland	Population-based prospective cohort study	781	5 (4 EATL, 1 DLBCL)	Risk of NHL significantly increased: SIR = 3.2 (95% CI 1.0–7.5). Increased mortality risk from lymphoproliferative diseases: SMR = 4.12 (95% CI 1.66–8.51)
Smedby et al. [47]	2006	Denmark and Sweden	Population-based case–control study	28	3055	Increased risk of: NHL: OR = 2.1 (95% CI 1.0–4.8) Diffuse large B-cell NHL: OR = 2.8 (95% CI 1.0–8.0) T-cell NHL: OR = 17 (95% CI 6.3–46)
Mearin et al. [48]	2006	10 European countries	Prospective, multicenter, case–control study	66	1446	Increased OR for NHL: 2.6 (95% CI 1.4–4.9)
Silano et al. [26]	2007	Italy	Population-based prospective cohort study	1968	20	Significant increase in NHL risk: SIR = 4.7 (95% CI 2.9–7.3)
Anderson et al. [30]	2007	United Kingdom	Population-based retrospective cohort study	490 (EMA+)	2	No significant increased risk of NHL despite a raised SIR [7.47 (95% CI 0.00–17.83)]
Goldacre et al. [31]	2008	United Kingdom	Hospital-based retrospective cohort	1997	11	Increased risk of NHL: adjusted Rate Ratio = 3.28 (95% CI 1.49–6.28). No significant increase in HL: adjusted Rate Ratio = 5.07 (95% CI 0.61–18.7)
Lohi et al. [37]	2009	Finland	Population-based retrospective cohort study	73 (EMA + subjects)	2	Increased mortality risk for lymphoma: RR = 9.51 (2.20–41.22)
Gao et al. [46]	2009	Sweden	Population-based case–control study	54 in NHL patients 7 in HL patients 40 in controls	37,869 NHL 8323 HL 236,408 controls	Increased risk of: NHL: OR = 5.35 (95% CI 3.56–8.06) HL: OR = 2.54 (95% CI 0.99– 6.56)
Anderson et al. [54]	2009	USA	Population-based case–control study	25	33,721	Borderline increased risk of NHL overall: OR = 1.5 (95% CI 0.9–2.5) Increased risk of: T-cell NHL: OR = 5.9 (95% CI 2.4–14) Marginal zone lymphoma: OR = 3.5 (95% CI 1.3–9.8)
Lohi et al. [32]	2009	Finland	Population-based retrospective cohort study	73 (EMA + patients)	2	Increased risk of lymphoproliferative diseases: RR = 5.94 (95% CI 1.41–25.04)
Grainge et al. [10]	2011	United Kingdom	Population-based prospective cohort study	1092	6	Increased risk of death from NHL: SMR = 7.06 (95% CI 2.59–15.4)
Elfstrom et al. [45]	2011	Sweden	Population-based retrospective cohort study	28,989 CD (Marsh 3) 13,140 inflammation (Marsh 1–2) 3711 positive serology	289	Increased risk of lymphoproliferative malignancy in: CeD patients: HR = 2.82 (95% CI 2.36–3.37) Inflammation: HR = 1.81 (95% CI 1.42–2.31) In patients with positive serology no increase in risk: HR = 0.97 (95% CI 0.44–2.14)
Grainge et al. [27]	2012	United Kingdom	Population-based retrospective cohort study	435	14	Increased risk of NHL: SIR = 12.0 (95% CI 6.55–20.1). The risk remained increased 15 years after CD diagnosis: SIR = 5.15 (95% CI 14.0–13.2)
Lebwohl et al. [55]	2013	Sweden	Population-based cohort study	7625	53	Increased risk of NHL: SIR = 2.81 (95% CI 2.10–3.67). In patients with persistent villous atrophy: SIR = 3.78 (95% CI 2.71–5.12) In patients with mucosal healing: SIR = 1.50 (95% CI 0.77–2.62)
Ilus et al. [56]	2014	Finland	Population-based prospective cohort study	32,439	132	Increased risk of NHL: SIR = 1.94 (95% CI 1.62–2.29) No increased risk of HL: SIR = 0.53 (95% CI 0.11–1.55) The SIR for NHL was increased (2.56) within 2 years from CeD diagnosis, but not at longer follow-up.
Abdul Sultan et al. [39]	2015	United Kingdom	Population-based retrospective cohort study	10,825	26	Mortality rate per 10,000 person years: 4.3 (2.9–6.3) in CeD vs. 1.4 (1.1–1.7) in controls. Patients with CeD had a 0.15% excess risk of dying from NHL up to 10 years post diagnosis.
van Gils et al. [49]	2018	Netherlands	Population-based, case–control study	261 in lymphomas and GI carcinomas 282 in controls (melanoma and basal cell carcinoma)	301,337 (lymphomas and GI carcinomas) 576,971 controls	Increased risk of T-cell lymphoma: RR = 35.8 (95% CI 27.1–47.4)
Quarpong et al. [34]	2019	United Kingdom	Population-based retrospective cohort study	602	16	SMR for lymphatic and hematopoietic malignancies: Overall = 5.16 (95% CI 2.95–8.38) Diagnosis at <15 years = 8.03 (95% CI 1.66–23) Diagnosis at ≥15 years = 4.77 (95% CI 2.54–8.16)
Koskinen et al. [40]	2020	Finland	Population-based cohort study	12,803	44	Increased risk of dying from lymphoproliferative diseases: HR = 2.36 (95% CI 1.65–3.39). HR decreased but remained significant after exclusion of the first 2 years of follow-up.
Lebwohl et al. [28]	2021	Sweden	Population-based cohort study	47,241	445 hematologic cancers 392 lymphoproliferative cancers	Increased risk of: Hematologic cancers: HR = 1.90 (95% CI 1.70–2.13). Lymphoproliferative cancers: HR = 2.20 (95% CI 1.94–2.49). Greater risk persists also excluding the first-year follow-up.

Abbreviations: CeD, celiac disease; NHL, non-Hodgkin lymphoma; HL, Hodgkin lymphoma; EATL, enteropathy-associated T-cell lymphoma; DLBCL, diffuse large B-cell lymphoma; HR, hazard ratio; SMR, standardized mortality ratio; RR, relative risk; aHR, adjusted hazard ratio; OR, odds ratio.
